# Microscopic characterization of Fe nanoparticles formed on SrTiO_3_(001) and SrTiO_3_(110) surfaces

**DOI:** 10.3762/bjnano.7.73

**Published:** 2016-06-07

**Authors:** Miyoko Tanaka

**Affiliations:** 1 Research Center for Advanced Measurement and Characterization, National Institute for Materials Science, Tsukuba, Ibaraki 305-0003, Japan

**Keywords:** epitaxial orientation relationship, iron (Fe), nanoparticles, morphology, scanning electron microscopy (SEM), strontium titanate (SrTiO_3_), transmission electron microscopy (TEM), Wulff construction

## Abstract

Fe nanoparticles grown on SrTiO_3_ (STO) {001} and {110} surfaces at room temperature have been studied in ultrahigh vacuum by means of transmission electron microscopy and scanning tunnelling microscopy. It was shown that some Fe nanoparticles grow epitaxially. They exhibit a modified Wulff shape: nanoparticles on STO {001} surfaces have truncated pyramid shapes while those on STO {110} surfaces have hexagonal shapes. From profile-view TEM images, approximate values of the adhesion energy of the nanoparticles for both shapes are obtained.

## Introduction

Metal nanoparticles on oxide substrates are one of the key materials in modern technology. Not only are they widely used in catalysis, there are also potential applications in nanoelectronics, spintronics, photonics, sensors, and fuel cells [1–5]. In these fields, particle morphology and interface structure are of utmost importance because they predominantly determine the properties of the material. Thus, a lot of effort is put into controlling them by adjusting, for instance, the compositions of substrates, reconstructions, surface patterns and polarities [6–10].

SrTiO_3_ (STO) is one of the substrate materials being extensively studied for these purposes. Attempts have been made to grow metal nanoparticles of controlled sizes and morphologies on its surface by using chemical etching, controlling geometry, fabricating reconstructions and buffer layers, and using ion bombardment [11–15]. For example, Silly et al. successfully controlled shapes of nanoparticles by tuning the reconstruction of a STO(001) substrate and the substrate temperature during deposition [12]. Sun et al. also controlled shapes of nanoparticles on a reconstructed STO(001) substrate and utilized them to catalyse carbon nanotubes [15]. However, since these studies image nanoparticles with STM, the exact interfacial structures, which affect the properties of the nanoparticles more than anything else, are not directly obtained. Since interface interaction significantly affects the size and morphology of a nanoparticle [16–18], cross sectional observation is greatly required for clarification of the exact interfacial structure.

At the same time, surface tuning in a less complicated and time-demanding way is necessary. One way of achieving this is to simply change the orientation of the substrate surface and use restrictions made by the crystal lattice. Metal films and nanoparticles of particular orientations were successfully fabricated by using this method [13,19–22]. In the present study, interfacial structures of Fe nanoparticles on STO(001) and STO(110) surfaces, their morphologies and arrangements are investigated by using a combined system for ultrahigh vacuum (UHV) transmission electron microscopy (TEM)/scanning tunnelling microscopy (STM). The system provides in situ observation of nanoparticles from both horizontal and vertical directions by combining STM plan-view observation and TEM plan-view and profile-view observations. The formation of the Fe/STO interface was achieved by depositing Fe nanoparticles on STO substrates that are annealed under UHV. The fabricated samples were transferred to the microscopes without exposing samples to air for further observation. Multi-angle observation reveals the interfacial structure along with their morphology. From analysis of profile-view TEM images, the approximate values of the adhesion energy of the nanoparticles for both shapes are obtained.

## Experimental

The experiments were performed in a UHV TEM/STM integrated characterization system (UTSICS), which is a combination of UHV sample preparation chambers and UHV microscopes (STM: JSPM-4500XT, TEM: JEM-2000VF) with a base pressure of ca. 10^−8^ Pa [23]. The specimens were prepared from SrTiO_3_(001) and SrTiO_3_(110) wafers (La-doped, 5 atom %). Bulk samples were used for STM observation, and ion-milled thin film samples for TEM observation. After chemical cleansing in NH_4_–HF buffer solution (BHF, pH ca. 4.5) for 5 min [24], they were annealed in the cleaning chamber of UTSICS at about 1100 K for 30 min by electron bombardment at 5 kV. Then they were examined either by STM or TEM to confirm the cleanliness of the surfaces.

Fe nanoparticles were deposited in a deposition chamber of the UTSICS using an electron beam at room temperature (RT). The deposition rate was about 0.01 nm/s. The coverage ranges from 0.5 to 2.0 nm. The vacuum during deposition was kept below 5 × 10^−8^ Pa. After deposition the samples were transferred to the microscopes again for further observation. The Fe nanoparticles were formed on both plan-view and profile-view surfaces [25]. Profile view surfaces are made at the very edge of the thinnest part of TEM samples. For a (001) substrate, the plan-view surface is the same STO(001) plane and the profile-view surfaces are perpendicular to this, typically STO(110) and STO(100) planes. For a STO(110) substrate, the plan-view surface is the (110) plane and the profile-view surfaces are typically (001) and (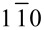) planes. Fe nanoparticles grown on plan-view surfaces are observed by STM and transmission electron diffraction (TED). Those on profile-view surfaces are observed by high-resolution TEM (HR-TEM).

STM images were taken in constant-current mode with a positive sample bias of 1.0–2.0 V. Electrochemically etched tungsten tips were used. STM images are not highly resolved because vibration and sample drift cannot be completely eliminated due to the relatively non-rigid sample holders that need to be compatible to UTSICS and TEM. TEM images and TED patterns were taken with an acceleration voltage of 200 kV. High-resolution images were acquired with a CCD camera (Gatan Orius SC200). The observation of the identical samples by both TEM and STM was not performed in the present study owing to experimental difficulties.

## Results and Discussion

Various surface structures are known to be formed on STO surfaces depending on preparation methods and conditions [26–40]. The influential factors are annealing temperature, ambient atmosphere, particularly the partial pressure of oxygen, and the conditions of surface sputtering before annealing. When the surface is sputter-cleaned, it becomes severely disordered, forming oxygen vacancies in the uppermost layers [28]. Subsequent annealing induces oxygen diffusion and surface recrystallization at the same time, making various superstructures possible to appear. In our experiments, TEM STO(001) substrates showed simple (1 × 1) structures [41]. This type of surface structure is reported for samples annealed in UHV at relatively low temperatures [26,33], which is also the case in our study. STO(110) surfaces are terminated with TiO planes with the possibility of oxygen depletion [25]. This type of surface structure is also reported with samples annealed in UHV at comparatively low temperatures [34–36] and matches with our results. However, crystal deformation presumably due to ion-beam thinning is also observed in our case [25].

**Figure 1:**
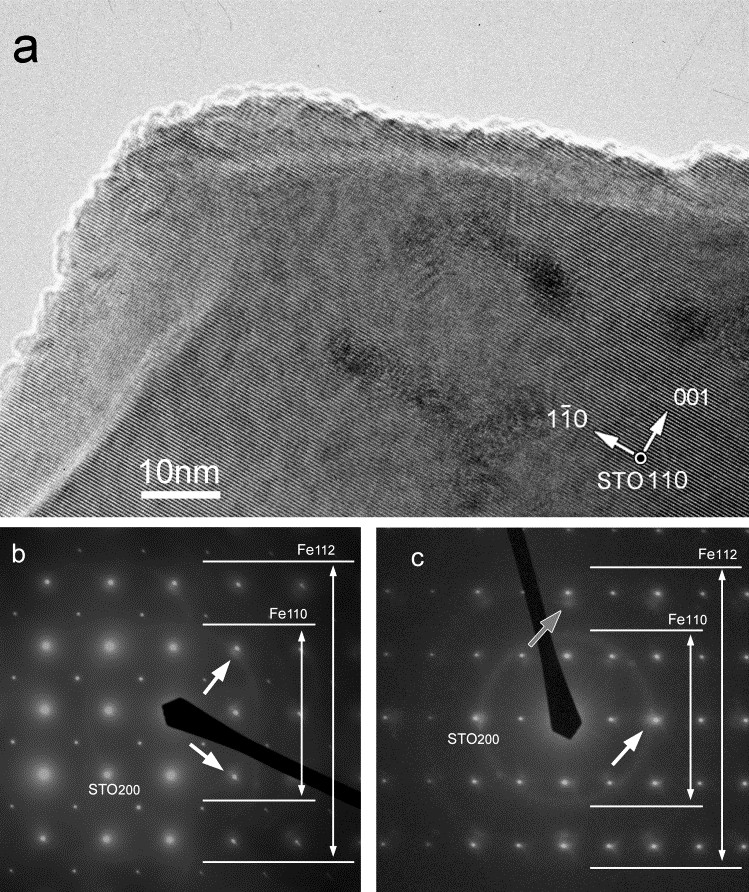
A TEM image and diffraction patterns of Fe nanoparticles on STO substrates. a) TEM plan-view image of Fe nanoparticles on STO(110); b,c) diffraction patterns taken from STO(001) and STO(110) substrates, respectively.

When Fe was deposited on these surfaces, Fe nanoparticles of similar sizes with several types of morphologies were formed. Figure 1 shows a TEM image (Figure 1a) and diffraction patterns from each substrate orientation (Figure 1b,c) after Fe deposition. From the TEM image that was taken of nanoparticles on a STO(110) substrate, the particle sizes were determined to be in the range of 2–3 nm. Nanoparticles on STO (001) substrates showed almost the same size range and density. In both diffraction patterns of a STO(001) substrate (Figure 1b) and of a STO(110) substrate (Figure 1c), faint Debye ring patterns that imply the formation of randomly oriented crystallites are recognizable. At the same time, there are a select number of regions of increased local intensity around the rings, as indicated by white arrows. Other arcs also exist (grey arrow in Figure 1c). These local increases in intensity denote that some of the nanoparticles grow epitaxially while others exhibit multiple orientations on the surface. Although iron diffusion on oxide surfaces is not well-quantified, it is assumed that iron atoms are not highly mobile during RT growth and that particles grow through direct adhesion of iron atoms to nuclei. When their interfaces are far from ideal low-index ones, the nanoparticles grow to irregular, random shapes. However, some of them happen to possess near-ideal interfaces to grow epitaxially with defined shapes. We focus on these epitaxially grown nanoparticles since these are the prototypes of higher temperature growth and we aim to form this type of nanoparticles in a controlled manner.

The orientation relationships of epitaxially grown nanoparticles on each substrate are


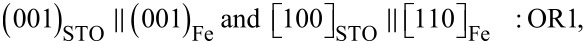



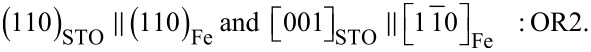


OR1 has been reported previously as “cube on cube” with 45° orientation [42–46], and is frequently promoted by a high density of coincident sites for bcc metal atoms on the O sub-lattice [47]. OR2 is a {110} plane version of OR1, namely, “rectangle on rectangle” with 90° orientation.

The deposition temperature (RT) for the present deposition rates is obviously below the epitaxial temperature, above which all nuclei have a single epitaxial orientation [48]. Although UHV environment and surface cleaning do contribute to lowering this temperature [49], RT was not sufficiently high for the epitaxial orientations to be uniquely defined. However, since OR1 and OR2 are the only clearly recognizable epitaxial relationships in the diffraction patterns, they can be regarded as the most favourable epitaxial orientation relationships for both substrates.

The formation of 3D particles, instead of a film, is expected on the basis of the surface free energies involved in the system. From the thermodynamic point of view, if

[1]
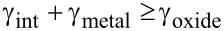


applies, where γ_int_, γ_metal_, and γ_oxide_ are the surfaces free energies of the metal–oxide interface, the metal, and the oxide, respectively, the metal is expected to form 3D aggregates (Volmer–Weber growth mode) [50]. From the previously reported surface free energies per unit area of the crystal facets [51–52], Equation 1 is supposed to hold well for the growth of a transition metal on an oxide substrate [53–55].

**Figure 2:**
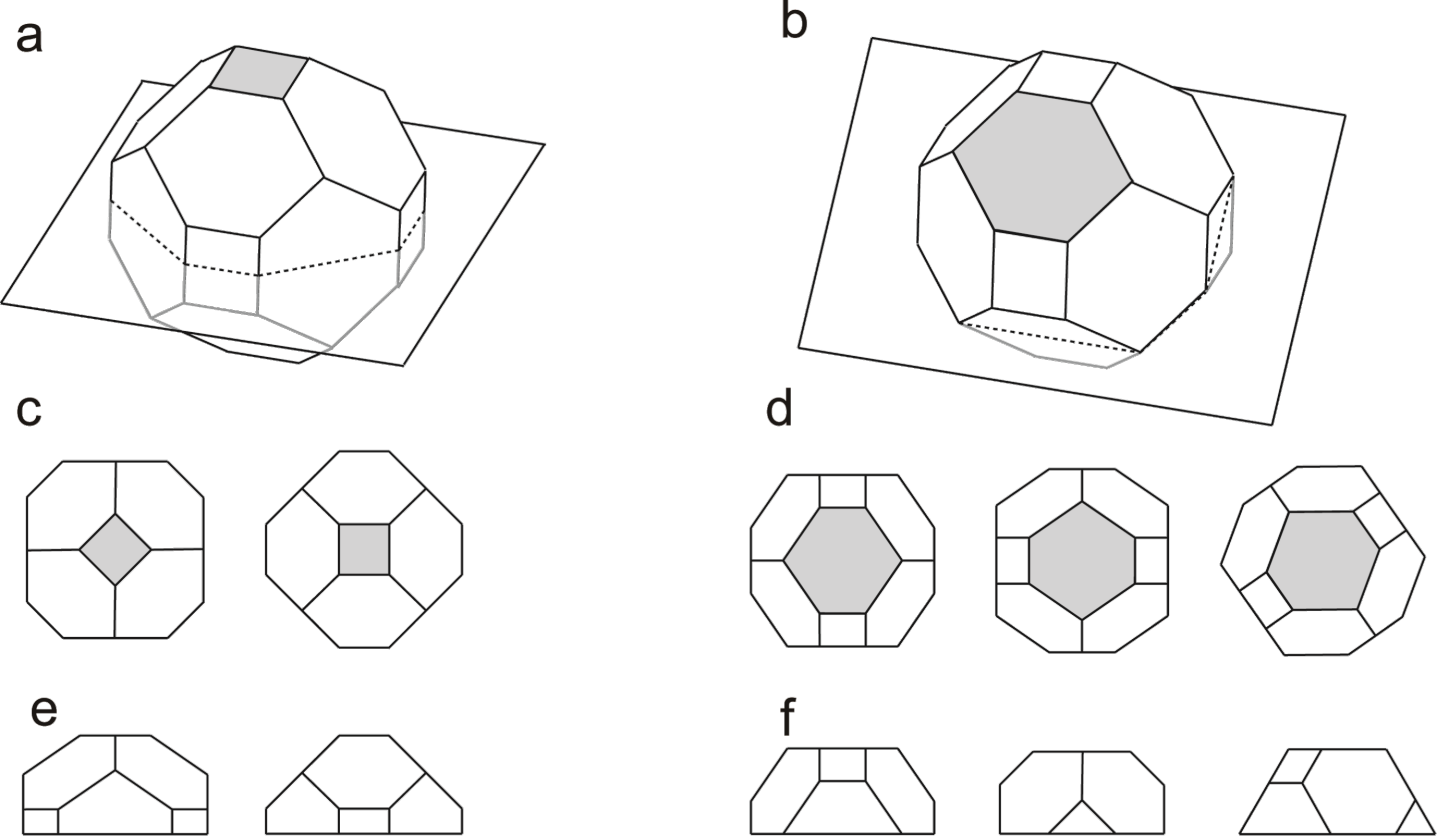
Schematic models of the nanoparticles with Wulff shapes. a) A truncated pyramid with OR1 and b) a distorted hexagon with OR2; c–f) corresponding top and side views. Top surfaces are shaded.

As mentioned above, there are nanoparticles with ideal interfaces. These nanoparticles may well be assumed to have nearly thermodynamically stable shapes, since surface self-diffusion lengths of iron atoms on both Fe(100) and (110) surfaces are reported to be larger than the nanoparticle sizes observed here [56–57]. Although a more precise evaluation of kinetic factors and of the possibility of anisotropic growth should be made, it is not unreasonable to suppose that ideal nanoparticles have a simple Wulff shape. And nanoparticles with OR1 and OR2 can be illustrated as shown in Figure 2. Figure 2a,b present the same schematic model being cut along different planes. They are drawn using only the least-energy surface and the second-least-energy surface, namely {110} and {100}, respectively. The surface free energy values are taken from the literature with γFe{110}/γFe{100} of 0.92 [51]. The nanoparticle in Figure 2a has Fe(001) top and bottom surfaces, and the nanoparticle in Figure 2b has Fe (110) top and bottom surfaces. Actual nanoparticles are located on a substrate so they do not possess a complete Wulff shape but are cut along a certain plane. This type of modified Wulff construction, which takes into account the particle–substrate interaction, is denominated as the Winterbottom construction [58]. The Winterbottom theory describes the dependence of the particle shape upon the anisotropy of the surface energy of the particle and upon the binding between the particle and the substrate. Figure 2c–f shows the top and side views of the Winterbottom constructions of the nanoparticles in Figure 2a,b. In the image the Wulff shapes are cut along the planes that contain the Wulff points (the centre of mass). Hereafter, we call nanoparticles with a configuration modelled in Figure 2c,d truncated pyramids and those in Figure 2e,f distorted hexagons.

**Figure 3:**
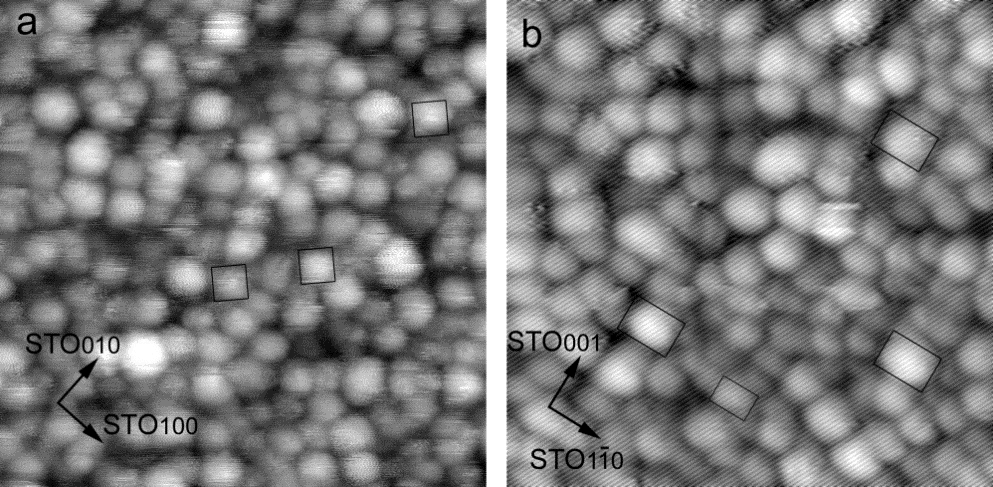
STM images of Fe nanoparticles on a) STO(001) (30.2 nm^2^, *V*_sample_ = +1.2 V, *I* = 0.2 nA), and b) STO(110) (21.5 nm^2^, *V*_sample_ = +1.5 V, *I* = 0.3 nA).

Since the precise contours of nanoparticles are not visualized by plan-view imaging due to their smallness, STM observation of Fe nanoparticles deposited on bulk substrates under exactly the same conditions were performed. After substrate annealing before Fe deposition, STM images of STO(001) and STO(110) surfaces showed (1 × 1) and (3 × 1) structures, respectively [33,38]. Both structures exhibit atomic disarrangement from place to place, which is attributed to oxygen depletion during UHV annealing by electron beam bombardment. Figure 3 shows STM images of the nanoparticles after deposition. Although the images are not highly resolved, nanoparticles with lateral sizes of 2–3 nm were imaged both for a STO(001) substrate (Figure 3a) and a STO(110) substrate (Figure 3b). The nanoparticles on a STO(001) substrate in Figure 3a mostly possess square or near square shapes. Many of them are aligned along either the 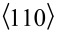 or the 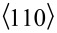 directions of STO (some of them are outlined in the image). These nanoparticles are most likely to have truncated-pyramid shapes and hence to have OR1 orientation. There are other nanoparticles with square shapes but differently oriented, or with distorted shapes. These are assumed to be of multiple orientations. The nanoparticles on a STO(110) substrate have more distorted shapes, and some of them are identified as rectangles or hexagons (outlined in the image). Some of these structures are aligned along the 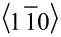 or the 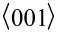 directions of STO and presumably have OR2 orientation. Other nanoparticles just have irregular shapes and are thought to exhibit multiple orientations.

**Figure 4:**
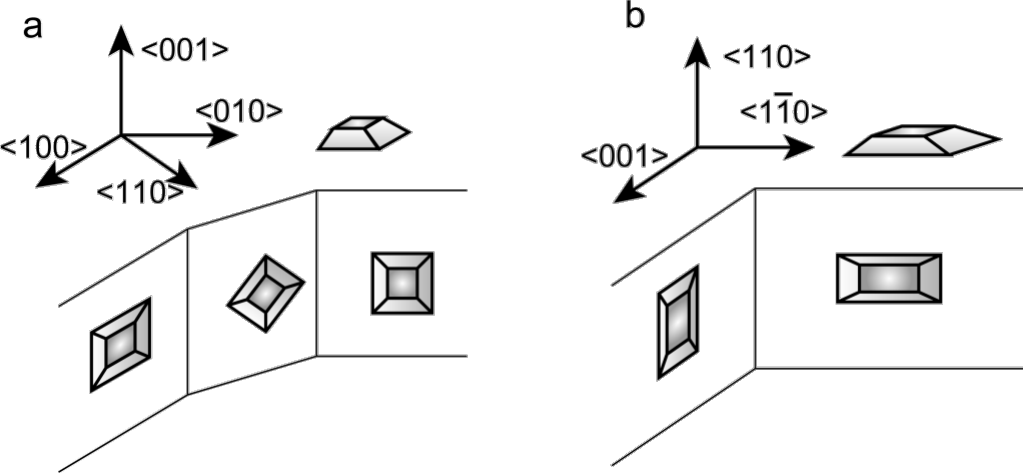
Schematic illustrations of profile-view imaging. a) Nanoparticles on STO{100} planes can be viewed from two directions that make a 45° angle, and b) those on STO{110} can be viewed from two directions that make a right angle.

HRTEM observation in a profile-view mode disclosed nanoparticles of major and of other orientations. Some of the nanoparticles deposited to a STO(001) substrate are located on the edge {100} or {110} planes with their low-index crystal planes perpendicular to the field of view. Likewise, some of the nanoparticles deposited on a STO(110) substrate are on the {001} or {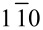} edge planes. Thus for the case of nanoparticles on STO{100} planes, they can be seen from two directions that make a 45° angle (schematically shown in Figure 4a), while those on STO{110} planes could be observed from two directions that cross at a right angle (Figure 4b).

**Figure 5:**
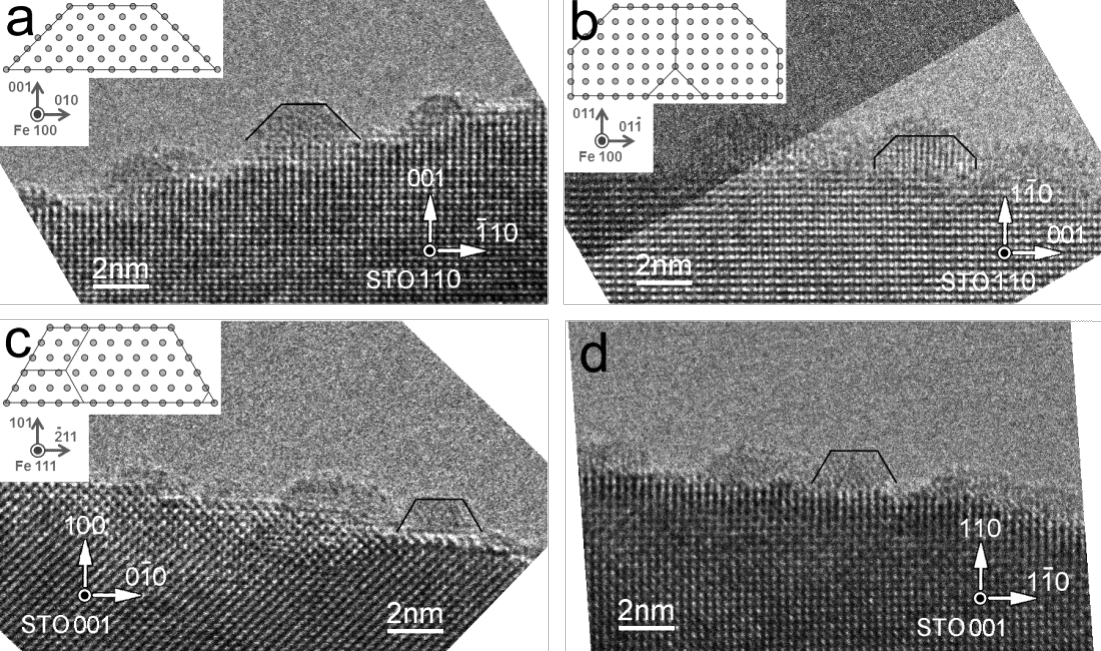
TEM profile-view images of Fe nanoparticles on substrate edges. a) Nanoparticles with OR1 orientation on STO (001); b) nanoparticles with OR2 orientation on STO (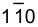). Nanoparticles with minor orientations on c) STO(100) and d)STO (110).

Nanoparticles observed from these directions are shown in the TEM images in Figure 5. In Figure 5a, some nanoparticles with OR1 orientation are seen on a sample edge. In the central nanoparticle, atomic planes of Fe{110}, which make 45° angles to the edge STO(001) surface, are observed. It is outlined by a black polygonal line for clarity. The inset shows an atomic model of a Winterbottom shape, namely a truncated pyramid. In Figure 5b, nanoparticles being viewed from a different angle are observed. Despite the unclear outlines due to nanoparticles overlapping in the vertical direction (perpendicular to the paper), some of them show clear Fe{110} planes that appear as the orthogonal lattice. This implies the formation of an interface with OR2 orientation. The inset shows a model corresponding to this shape, namely distorted hexagon (hexagon1; hex1). The spacing of Fe{110} planes and the substrate STO{002} planes are 0.202 and 0.195 nm, respectively. Hence, the nanoparticles are under a compressive stress of about 3.6%. Closer look at the outlined nanoparticle reveals the gradual widening of atomic planes from the interface to the top surface to release compressive stress.

Nanoparticles with other epitaxial orientations were also observed. Figure 5c,d shows nanoparticles with their {110} planes parallel to the interfaces. In each image, one of the nanoparticles, which is outlined by a black polygonal line, has this configuration. The oblique parts of the lines make 60° angles to the interface. Both nanoparticles are showing the same Fe{111} planes perpendicular to the paper, although the nanoparticle in Figure 5c sits on a STO{100} surface and the one in Figure 4d on a STO{110} surface. Thus, the model shown in the inset in Figure 5c (hexagon2; hex2), which shows a distorted hexagon viewed from a different angle from the one in Figure 2b, applies for both nanoparticles. It is intriguing that distorted hexagons could be placed on different substrate planes and/or with different surface orientations. Since they have the least-energy Fe{110} interface planes, it is assumed that this morphology is preferred to minimize its total energy and that interface matching is less important in this case. Kamaratos et al. reported that no iron oxidation at the metal–oxide interface was indicated in their adsorption study of Fe on the STO(001) surfaces [46]. This may explain the priority of nanoparticles to surface energies than interface matching in some cases.

The shape of nanoparticles at surfaces is determined by the Wulff–Kaishev construction [59] such as

[2]
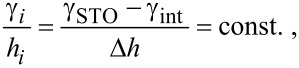


where *h_i_* is the distance from the Wulff point to each facet, *Δh* is the distance from the Wulff point to the interface, *γ_i_* is the surface free energy of the nanoparticle facet *i*, *γ*_STO_ is that of the STO substrate, and *γ*_int_ is the interfacial free energy, respectively. Since *h_i_* can be expressed in terms of geometrical factors such as height-to-length (*h*/*l*) ratios, the value of *Δ*γ *≡ γ*_STO_ − *γ*_int_ for each nanoparticle shape can be obtained as

[3]
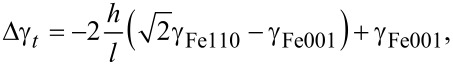


[4]
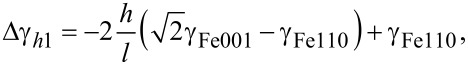


[5]
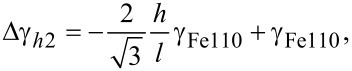


for truncated pyramids, distorted hexagons seen from Fe{100} (hex1) and Fe{111} (hex2) directions, respectively. The adhesion energy γ_adh_ is defined by

[6]
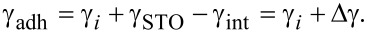


The height-to-length ratios are obtained from our profile images as ca. 0.75, ca. 0.60, and ca. 0.57 for pyramids, hex1, and hex2, respectively. Although the limited number of nanoparticles with low-index planes prevents a statistical analysis. Using these values and additional literature values [51] in Equations 3–5 result in γ_adh_ of ca. 3.95, ca. 3.23, and ca. 3.37 for pyramids, hex1, and hex2, respectively. It should be noted that this estimation does not take into account the surface conditions, especially the presence of oxygen vacancies formed during UHV annealing. Our previous studies found that surfaces of both STO(001) and STO(110) have oxygen-depleted (1 × 1) structures [25]. Surface energies for these may differ from those of fully oxidized ones and may alter above values. Still, the present estimation provides a guide for energy differences depending on nanoparticle morphologies, and can be applied for those grown on substrates prepared under reducing conditions.

Hex1 and hex2 have almost the same adhesion energy, since they have the same hexagonal shape but only viewed from different directions. The adhesion energy of nanoparticles with major orientation relationship (pyramid) is larger than that of minor orientation relationship (hexagon). This explains why truncated pyramids seem flatter. This is reasonable taking into account the facts that the interface atoms of the interface with major orientation match better, and that OR1 is thought to be thermodynamically stable [45].

The formation of minor epitaxial relationships (hexagons) might originate from surface imperfections [60–61]. If defects are present on the surface, the deposited atoms may be trapped at these sites forming nuclei for subsequent nanoparticle growth. Adjacent nuclei growing independently may merge and grow into a larger nanoparticle with a non-equilibrium interface. Charge effects should also be considered [62–63]. Both STO(001) and STO(110) surfaces could exhibit variations of charge density associated with local depletion of O^−^, which was caused during sample preparation by ion-beam bombardment and UHV annealing. These surface charges, which induce an electric field variation at the interface, could affect the local interfacial reactivity [64–65] and hence the position of each deposited atom. It is also possible that there exist metastable interfaces [66] or a dependence of the interface structure on the size and the shape of nanoparticles [67].

Of particular interest is that nanoparticles with certain morphologies and interfaces preferentially grow on STO(110) surfaces. We have previously found a rectangle-on-rectangle relationship for fcc Ni on STO(110) surfaces, in which case the relation is (110)_STO_ 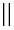 (110)_Ni_ and [001]_STO_ 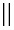 [001]_Ni_ [25]. This epitaxy is more intuitive since the configuration is a tilted version of the well-known cube-on-cube relationship [47], and rectangular atomic lattices match in the way that the long and the short sides align together. In the present case, however, OR1 is not a “proper” cube-on-cube but one with 45° orientation. Tilting it yields the rectangle-on-rectangle 90° orientation. This may hint that rectangular lattices of oxide surfaces favour rectangular interfaces, contrary to what has been found for the deposition of metals on metal surfaces [68]. The previous and the present results suggest the possibility of selective growth of nanoparticles with rectangular basis.

## Conclusion

Fe nanoparticles grown on SrTiO_3_{001} and {110} surfaces were studied using a UHV–STM/TEM combined system. The surfaces were annealed by electron beam irradiation in UHV before deposition. Oxygen-depleted surface structures were formed for both TEM and STM substrates. Some nanoparticles grow epitaxially on both substrates. They have a single major epitaxial orientation relationship on each substrate. The one on STO(001) surfaces was the cube-on-cube with 45° orientation, namely (001)_STO_ 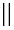 (001)_Fe_ and [100]_STO_ 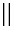 [110]_Fe_. On the other hand, the one on STO(110) surfaces was a tilted version of this such that (110)_STO_ 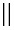 (110)_Fe_ and [001]_STO_ 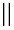 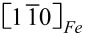. Nanoparticles with the former epitaxy exhibit the shape of a truncated pyramid while those with the latter epitaxy exhibit a hexagonal shape. Profile-view TEM observation revealed their height-to-length ratios, which in turn give the approximate value of the adhesion energy of each shape. The preference of each substrate for certain epitaxial orientation and morphology is described thoroughly.
